# Investigations of the prevalence and virulence of *Candida albicans* in periodontal and endodontic lesions in diabetic and normoglycemic patients

**DOI:** 10.1590/1678-7757-2016-0432

**Published:** 2017

**Authors:** Cinthya Cristina GOMES, Ludmila Silva GUIMARÃES, Larissa Christina Costa PINTO, Gabriela Alessandra da Cruz Galhardo CAMARGO, Maria Isabel Bastos VALENTE, Maria Inêz de Moura SARQUIS

**Affiliations:** 1 Universidade Federal Fluminense, Faculdade de Odontologia, Departamento de Formação Específica, Nova Friburgo, RJ, Brasil.; 2 Universidade Federal Fluminense, Faculdade de Odontologia, Programa de Pós-graduação em Odontologia, Nova Friburgo, RJ, Brasil.; 3 Instituto Osvaldo Cruz, Departamento de Micologia, Laboratório de Taxonomia, Bioquímica e Prospecção de Fungos, Rio de Janeiro, RJ, Brasil.

**Keywords:** Infection, Candida albicans, Diabetes mellitus

## Abstract

Pulpal and periodontal tissues have similar microbiota that allows cross-contamination between the pulp and periodontal tissues.

**Objective:**

The aim of this study was to investigate the prevalence of isolated *Candida albicans* from periodontal endodontic lesions in diabetic and normoglycemic patients, and the fungi's virulence in different atmospheric conditions.

**Material and Methods:**

A case-control study was conducted on 15 patients with type 2 diabetes mellitus (G1) and 15 non-diabetics (G2) with periodontal endodontic lesions. Samples of root canals and periodontal pockets were plated on CHROMagar for later identification by polymerase chain reaction (PCR) and virulence test.

**Results:**

*C. albicans* was identified in 79.2% and 20.8% of the 60 samples collected from diabetic and normoglycemic patients, respectively. Of the 30 samples collected from periodontal pockets, 13 showed a positive culture for *C. albicans*, with 77% belonging to G1 and 23% to G2. Of the 11 positive samples from root canals, 82% were from G1 and 18% from G2. Production of proteinase presented a precipitation zone Pz<0.63 of 100% in G1 and 72% in G2, in redox and negative (Pz=1), under anaerobic conditions in both groups. Hydrophobicity of the strains from G1 indicated 16.4% with low, 19.3% with moderate, and 64.3% with high hydrophobicity in redox. In G2, 42.2% had low, 39.8% had moderate, 18% had high hydrophobicity in redox. In anaerobic conditions, G1 showed 15.2% with low, 12.8% with moderate, and 72% with high hydrophobicity; in G2, 33.6% had low, 28.8% had moderate, and 37.6% had high hydrophobicity. There was statistical difference in the number of positive cultures between G1 and G2 (p<0.05) with predominance in G1. There was statistical difference for all virulence factors, except hemolysis (p=0.001).

**Conclusions:**

*Candida albicans* was isolated more frequently and had higher virulence in diabetic patients.

## Introduction

Combined periodontal/endodontic lesions have been characterized as infectious processes that occur simultaneously in the pulp and the periodontal tissue of the same tooth^1^.

Dental pulp and periodontal tissues have similarities regarding microbiota and fungi infections in root canals and advanced periodontitis_7,9_. In the oral cavity, fungi can be isolated from mucosal surfaces, however, it can also be found in biofilm, root canal infections, peri-implant lesions, and periodontal pockets^24^. *Candida albicans* is a commonly identified species of fungi and has higher prevalence in saliva and in the root canal^6^. It is considered an opportunist microorganism found not only in teeth with pulp necrosis and apical periodontitis^8^, but also in periodontal pockets, suggesting that it may be involved in the pathogenesis of periodontal disease^5^. *Candida* in the oral cavities could facilitate the increase of pathogenic microorganisms as it modifies the host defense mechanism. There is a correlation between increased incidence of infections caused by fungi and immunocompromised patients, including patients with hematological diseases, AIDS, diabetes mellitus, broad spectrum antibiotics users, and those that use steroids in high doses^5,8,24^.

Diabetes mellitus is a chronic disease that may reduce resistance to microbial infection of tissues and decrease tissue repair capacity_27_. It is both an endocrine and a metabolic dysfunction involving the control of blood glucose levels, resulting in hyperglycemia. Chronic hyperglycemia may trigger deficiencies in the immune system, increasing the risk of infections, including in the oral cavity^14,15^.

An association between diabetes and periodontal disease has been well documented and poor glycemic control may lead to increased severity of periodontitis_14_. A bidirectional relationship between diabetes mellitus and periodontal diseases has been discussed. It is estimated that patients with poor glycemic control are three times more likely to develop periodontal destruction compared to normoglycemic individuals^14^. However, the literature on pathogenesis, progression, and endodontic treatment of pathologies in diabetic patients is extremely sparse_7_.

*Candida* species possess multiple virulence factors that help in the invasion of the host tissue and evasion of host defence mechanisms. Such factors include hydrolitic enzymes production, hydrophobic interaction, hemolytic activity. The hydrolytic enzymes include secreted aspartyl proteinase (SAP) and phospholipase that cause the inhibition of fagocitosis processes by degrading immunoglobulins and extracellular matrix proteins^18^. Hydrophobic interaction play an important role in the adherence of pathogenic microorganisms to host cells by facilitating contact between the parasite and host cell^19^. Hemolysin activity, followed by iron acquisition, is considered an important virulence attribute of *Candida* species. Iron is used by the fungus for metabolism, growth and for facilitating hyphal invasion during host infection^17^. The hemolysin activity of *C. albicans* in diabetic patients may be directly or indirectly increased in the presence of high glucose concentration in the blood^25^.

The aim of this study was to assess the prevalence of *Candida albicans* isolated from periodontal endodontic lesions of diabetic and nondiabetic patients. Additionally, some virulence factors of *Candida albicans* were evaluated, such as activity of the proteinase, phospholipase, and hemolysin, and hydrophobicity in a reduced oxygen atmosphere or in anaerobic conditions.

## Material and methods

This research was approved and is in accordance with the Ethical Committee for Research through the document CEP number 125.285, CAAE number 04247712.3.0000.5243 and Clinical Trials identification NCT02867254. All procedures performed in this study were in accordance with the 1964 Helsinki declaration. Additional informed consent was signed by all participants for whom identifying information is included in this paper. This case-control study was conducted on thirty patients (15 patients with type 2 diabetes mellitus and 15 normoglycemics) with clinical and radiographic diagnosis of a periodontal endodontic lesion. To assess the glycemic control of the patients, plasma glucose level and glycosylated hemoglobin concentrations (HbA1c) were measured. Plasma glucose levels of each patient at the moment of sampling was not estimated. The diabetes diagnosis was performed according to the Guidelines of The American Diabetes Association, which recommends the measurement of plasma glucose and glycosylated hemoglobin level concentrations (HbA1c)^2^.

All patients were initially submitted to examination to verify the clinical periodontal parameters (plaque index, probing bleeding, probing depth, clinical attachment level, and gingival recession) and pulp sensitivity testing. The study included patients who presented periodontal pockets involving the apical region of the tooth and pulp necrosis (periodontal endodontic lesion). Exclusion criteria were: use of antibiotics in the last six months, pregnancy, smoking, other systemic diseases, clinical signs of oral candidiasis, and patients who did not sign a free and clarified consent term.

### Sample collection

The teeth were isolated with a rubber dam isolation. The crown and surrounding structures were disinfected with 5.25% sodium hypochlorite, and neutralized with 5% sodium thiosulfate. The sterility of the external surfaces of the crown was checked by taking a swab sample from the crown surface and streaking it on blood agar plates, which were then incubated both aerobically and anaerobically. The preparation of the access cavity was made using a sterile diamond drill speed, without the use of water spray, but with manual irrigation with sterile saline. Before entering the pulp chamber, the access cavity was again disinfected according to the protocol described above. The sterility of the internal surface of the access cavity was checked as previously described, and all procedures were performed aseptically. Sampling of the periodontal pocket was made at the deepest site through three sterile paper points, inserted one by one for one minute. Prior to periodontal sampling, all participants were requested to rinse their mouth with 10 ml of 0.12% chlorhexidine gluconate solution (Periogard^®^ – Colgate-Palmolive Company, São Paulo, SP, Brazil), and the local area was dried and isolated with cotton rolls. Material collected from the periodontal pocket and the root canal was inoculated in Eppendorf tubes containing 1 ml of reduced transport fluid (RTF). Subsequently, samples from each site were diluted and plated on the medium Saboraund Dextrose Agar plus Chloramphenicol and chromogenic medium (CHROMagar Candida^®^, BioMerieux, Rio de Janeiro, RJ, Brazil) and incubated at 37°C for 48 h in a reduced oxygen atmosphere (10% CO_2_ and 90% air). The green colonies that grew on CHROMagar plates were chosen at random and stored in glycerol at 20°C for later identification by PCR.

### Molecular characterization of the species

Fifty ml of Sabouraud medium were inoculated with conidia and incubated for 72 h at 300°C. Mycelium was transferred to a 50 ml polypropylene screw-capped bottle containing six glass beads (4 mm in diameter). The tube was immersed in liquid nitrogen for 10 s and stirred vigorously with a vortex mixer for 20 s. Then, 2 ml of DNA extraction buffer and 0.5 mg per ml proteinase K (Sigma Chemical Co., St. Louis, MO, USA) were added, and the mixture was allowed to thaw. For the next step, the mixture was incubated for 2 h at 55°C. Proteinase K was inactivated by heating the mixture at 950°C for 10 min. A volume of 0.7 ml of the mixture was transferred to a 1.5 ml microfuge tube and an equal volume of phenol-chloroform (1:1) was added. The mixture was centrifuged at 10.000x g for 5 min at 40°C. Supernatant was transferred to a new tube and the same procedure was repeated using chloroform isoamyl alcohol (24:1). DNA was precipitated with 2 volumes of ethanol at -200°C and centrifuged at 12.000 x g for 20 min at 40°C. The pellet was allowed to dry. After washing with 70% ethanol at 40°C, the extracted DNA was dissolved in 50 μl of distilled water and 5 μl of suspension and was used for PCR.

### Polymerase chain reaction (PCR)

PCR reactions were standardized for each primer, using genomic DNA strains as a positive control. PCR reaction with DNA was performed for each universal primer to verify the presence of DNA as *C. albicans* (Forward: 5 ‘-ACT GCT CAA ACC ATC TCT GG-3 ‘ and Reverse: 5 ‘ -CAC AAG GCA AAT GAA GGA AT-3; with fragment size of 472 bp)^19^, and sterile distilled water was used as a negative control. Amplification was performed in T100 Thermal Cycler (PowerPac Basic, Bio-Rad Laboratories LTDA, Santo Amaro, SP, Brazil), programmed according to the following steps: pre-denaturation (94°C/5 min, 30 cycles), denaturation (94°C/30 s), annealing (TaoC calculated/30 s), and extension (72°C/1 min 30 s). Final extension was performed at 72°C for 4 min^20^.

### Electrophoresis of amplification product

After amplification, an analiquot of 2 μl race marker “Low DNA Mass Ladder” or “123 ladder (Invitrogen)” was added along with an equal volume of glycerol race containing dye [bromophenol blue 0.25% (w/v), xylenes cyanol FF 0.25% (w/v), 30% glycerol (w/v) in water], and the total volume was applied to a channel of the gel. An aliquot of 10 μl of PCR reaction product was added to the DNA 5 μl at a time separately from the running dye. Dilutions were applied on different channels and subjected to electrophoresis (PowerPac Basic, Bio-Rad Laboratories LTDA, Santo Amaro, SP, Brazil) in agarose gel at 1.5% (w/v) in TAE 1X buffer (Tris-acetate, 40 mM EDTA 1 mM, pH 8.0) at 90 V for 90 minutes. The gel was subsequently treated with ethidium bromide at 0.5 μl/ml and DNA was visualized with the aid of an ultraviolet transilluminator (UV) and photographed with the aid of an automatic detection and image analysis system (Gel Doc XR + System with ImageLab Software, Bio-Rad Laboratories LTDA, Santo Amaro, SP, Brazil).

### Proteinase and phospholipase activity determination

All isolates of *C. albicans* were tested in triplicate, for three separate experiments, to verify the enzymatic activity of proteinases (SAPS) and phospholipases^16,28^. The proteinase culture medium was BSA (bovine serum albumin) and the phospholipase culture medium consisted of enriched egg yolk. Plates were incubated at 37°C in a reduced oxygen atmosphere or in anaerobic conditions for 48 h to examine the proteases and for 72 h to examine phospholipases. Enzymatic activity was determined by the formation of a halo around the colony of yeast. The halo was measured in terms of the relationship between the diameter of the colony and the overall diameter of the colony plus the precipitation zone (Pz), according to the method described by Price, Wilkinson and Gentry^16^ (1982).

### Determination of hemolysin activity

Hemolysin activity was evaluated for all the isolates of *C. albicans* using blood agar medium^25^. Samples were grown on SDA for 24 h and incubated at 37°C in either a reduced oxygen atmosphere or anaerobic conditions for 48 and 72 h, respectively. An additional 10 μl of saline without yeast was placed on the same plate. The reference strain of *C. albicans* (ATCC 90.028) was used as a positive control. In addition, standard strains of *Staphylococcus aureus* (ATCC 10.832) and *Streptococcus mutans* (UA159), which repectively induce hemolysis beta and alpha, were used as a positive control. Gamma-hemolytic samples produce no halo, alpha-hemolytic samples produce a green halo, and beta-hemolytic samples produce a yellow halo. Assays were also conducted in triplicate.

### Cellular superficial hydrophobicity assay (CSH)

This assay was performed according to Rodrigues, et al.^19^ (1999) for all isolates of *C. albicans,* in two independent experiments. Fifty ml of Sabouraud dextrose broth (SDB, Disco Laboratories Detroit, MI, USA) were inoculated with yeast cells and incubated overnight at 37°C in a 10% reduced oxygen environment or in anaerobic conditions. Yeast cells were harvested and washed twice in phosphate buffer (pH 8.0). Yeast slurry was prepared in the same buffer to reach an optical density [(A0) 0.4 - 0.6 at 600 nm], and 150 μl of hexadecane was added to 3 ml of this yeast suspension. After 10 min incubation at 30°C, tubes were agitated by vortexing twice for 30 seconds. After allowing phase separation for 30 min, the optical density of the lower aqueous phase (A1) was measured and compared with the optical density obtained before the mixing step (A0). The percentage of cells in the hexadecane layer (adherent cells) was used to estimate the hydrophobicity, using the following formula: %H=A0-A1/A0 x 100%.

### Statistical analysis

Data distributions were expressed as means, standard deviations (SD), ranges and percentages, as appropriate. The results obtained for the tested virulence factors were analyzed according to the atmospheric condition, using Chi-square and Wilcoxon nonparametric tests. Differences were considered statistically signiﬁcant when p<0.05. All data were analyzed using the Statistical Package for Social Sciences (SPSS, Chicago, IL) version 16.0.

## Results

Diabetic and control groups were homogenous. Mean and standard deviation were obtained to establish comparisons between groups (Table 1).

**Table 1:** Main characteristics of the subjects studied

	Diabetics (n=15)				Controls (n=15)			
	Min	Max	Mean	SD	Min	Max	Mean	SD
age	38	69	52	10.23	39	70	53.3	12.12
PD	4	14	8.2	3	4	14	8.3	3.2
CAL	9	18	13.5	3.34	7	24	12.9	4.44
HBA1C	6.2	11.3	8.5	1.78	5	5	5	0
GLIC_L	27	295.8	190.3	71.64	74	90	84.5	5.01
		Diabetics (n=15)				Controls (n=15)		
			n (%)				n (%)	
Sex		male	6 (40)			male	5(33.3)	
		female	9 (60)			female	10 (66.7)	
PI								
		no	0			no	1 (6.7)	
		yes	15 (100)			yes	14 (93.3)	
GI								
		no	1 (6.7)			no	2 (13.3)	
		yes	14 (93.3)			yes	13 (86.7)	

PD, probing depth; CAL, clinical attachment loss; HBA1C, glycosylated hemoglobin concentrations; GLIC, plasma glucose level; PI, plaque index; GI, gengival index

Of the 60 samples collected (Figures 1 and 2) – 30 from periodontal pockets and 30 from root canals – 24 (40%) showed a positive culture for *C. albicans*. Nineteen of these 24 were from patients with diabetes mellitus and five from normoglycemics, corresponding to 79.2% and 20.8% of the collected samples, respectively. Of the 30 samples collected from periodontal pockets, 13 (43.3%) had a positive culture, 10 belonged to diabetic patients (77%) and 3 were from normoglycemics (23%). Of the 11 positive samples from root canals, 9 were from patients with diabetes mellitus (82%) and 2 from normoglycemic patients (18%).

**Figure 1:**
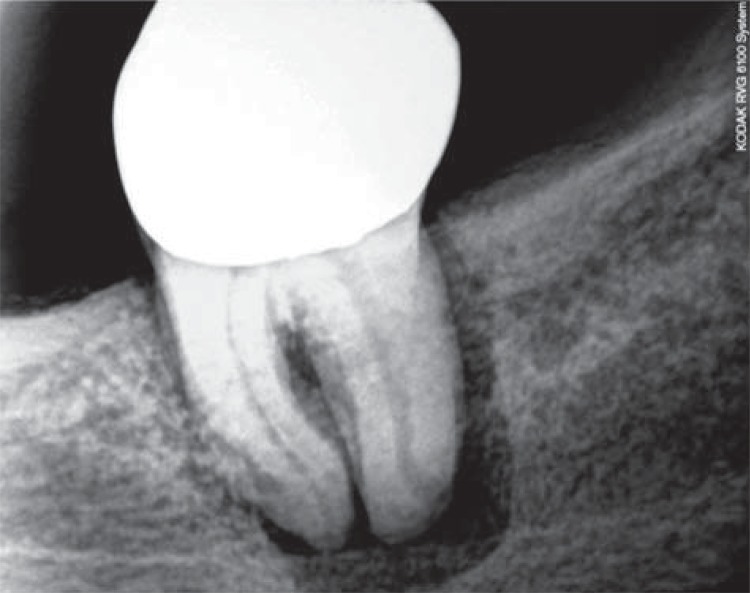
Radiographic image of tooth with periodontal endodontic lesion

**Figure 2:**
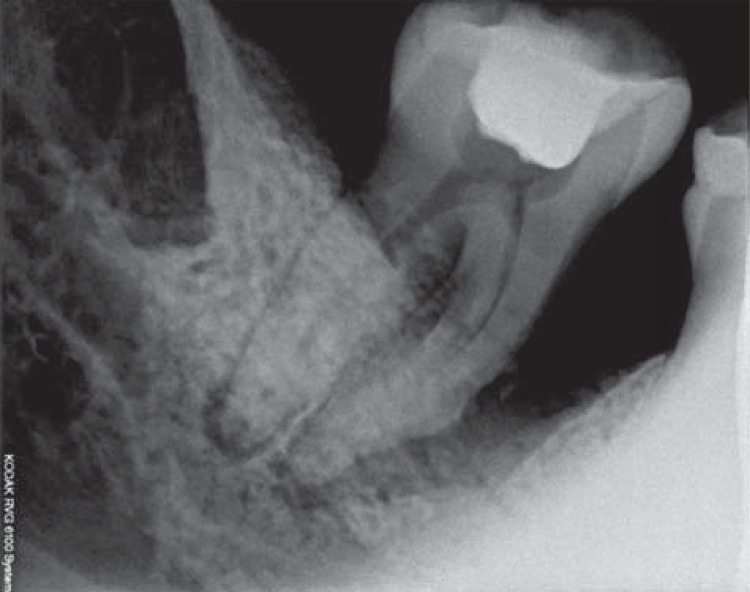
Radiographic image of tooth with periodontal endodontic lesion

*C. albicans* strains were found more often in diabetic patients. There was a statistical difference for the number of strains isolated from periodontal pockets and root canals of diabetic and normoglycemic patients when analyzed by Chi-square test (p<0.05).

Strains of *C. albicans* showed an increased virulence in diabetic patients (Table 2), demonstrating a statistically significant difference (p=0.001) after applying the Wilcoxon test. Hemolysin activity was positive for both groups in both atmospheres, but was not significantly different among test groups in both atmospheres.

**Table 2:** Virulence of *Candida albicans* strains

Virulence of *Candida albicans* strains	Diabetic (G1)	Normoglycemic (G2)
	Redox Anaerobic	Redox Anaerobic
	Negative (Pz=1) 0 100%	Negative (Pz=1) 2% 100%
PROTEINASE	Positive (Pz=1 - 0.63) 0 0	Positive (Pz=1 - 0.63) 6% 0
	Strongly Positive 100% 0 (Pz<0.63)	Strongly Positive 72% 0 (Pz<0.63)
	Redox Anaerobic	Redox Anaerobic
	Negative (Pz=1) 18.5% 51.2%	Negative (Pz=1) 0.3% 100%
PHOSPHOLIPASE	Positive (Pz=1 - 0.63) 28.3% 15.3%	Positive (Pz=1 - 0.63) 6.4% 0
	Strongly Positive 53.2% 33.5% (Pz<0.63)	Strongly Positive 23.3% 0 (Pz<0.63)
	Redox Anaerobic	Redox Anaerobic
	Alpha 84.8% 80.4%	Alpha 81% 70.3%
HEMOLYSIN	Beta 15.2% 19.6%	Beta 19% 29.7%
	Gama 0 0	Gama 0 0
	Redox Anaerobic	Redox Anaerobic
	Low 16.4% 15.2%	Low 42.2% 33.6%
HYDROPHOBICITY	Moderate 19.3% 12.8%	Moderate 39.8% 28.8%
	High 64.3% 72%	High 18% 37.6%

Production of proteinase presented Pz<0.63 of 100% in G1 and 72% in G2 in redox and negative (Pz=1), under anaerobic conditions in both groups. Phospholipase activity was higher in samples from G1 in redox and anaerobic conditions. Hydrophobicity of the strains of G1 indicated 16.4% with low, 19.3% with moderate and 64.3% with high hydrophobicity in redox. In G2, 42.2% showed low, 39.8% moderate, 18% high hydrophobicity in redox. Anaerobically, G1 presented 15.2% with low, 12.8% with moderate and 72% with high hydrophobicity; G2, showed 33.6% with low, 28.8% with moderate and 37.6% with high hydrophobicity. There was statistical difference in the number of positive cultures between G1 and G2 (p<0.05) with predominance in G1. There was statistical difference for all virulence factors, except hemolysis (p=0.001).

## Discussion

Fungi are considered normal inhabitants of the oral cavity, however, it may cause diseases when local or systemic factors such as diabetes mellitus predispose the individual. Premkumar, Ramani and Chandrasekar^15^ (2014) showed that patients with diabetes mellitus have a significantly increased prevalence of *C. albicans* strains in the oral cavity. The prevalence rate of the *Candida* species in diabetics was 87.5%, whereas in the control subjects it was 50% (collected by oral rinse method). In the present study, 40% showed a positive culture for *C. albicans*, 79.2% were from patients with diabetes mellitus, and 20.8% from normoglycemics. This discrepancy may be explained by the fact that the samples in this study were collected from periodontal pockets and root canals.

*Candida albicans* is the fungal species most commonly detected in the oral cavity, both in healthy individuals and in individuals with systemic involvement^23^. It is also the most often isolated fungal species in chronic periodontitis^19,21^ and infected root canals^22,23^. Kumar, Muralidhar and Banerjee^11^ (2015) isolated *C. albicans* in 21% of samples from patients with periodontal disease. Urzua, et al.^26^ (2008) isolated the fungus in 69.2% of periodontal sites of chronic periodontitis. In the present study, *C. albicans* was isolated in 43.3% of the samples collected from the periodontal pockets. Different results could be related to either different collection sites or systemic factors, as these samples were composed by both diabetic and normoglycemic patients.

This higher incidence of *C. albicans* in diabetic patients may be explained by the alteration of the normal oral flora in response to endocrine disorders of diabetes mellitus. Moreover, it may be attributed to increased adhesion of fungi in epithelial cells facilitated by increased glucose content in saliva, genetic susceptibility to infection, changes in defense mechanisms, and changes in local factors, including disadvantaged blood support^1^.

However, BremenKamp, et al.^4^ (2011) showed no significant difference in the prevalence of *C. albicans* between diabetic and normoglycemic patients. This contradiction with our results can be attributed to the fact that patients included in the study cited above beared glycated haemoglobin levels lower or equal to 9%, whereas in the present study patients presented glycated hemoglobin levels ranging from 6.2% to 11.3%. Blood glucose levels have a positive relationship with increasing transport rate and cell density in diabetic patients^3^.

Virulence factors of the *C. albicans* species may be involved in the pathogenesis of various oral diseases. However, few studies have investigated its role in host colonization and development of infections^10^.

In this study, it was observed that the fungi present in diabetic patients had proteinase, phospholipase, and hemolysin activity, and cell surface hydrophobicity in both tested environments. This is in line with the literature on this fungi’s adaptive survival conditions^12^.

In the present investigation, proteinase expression was detected in 100% of the strains of *C. albicans* when incubated in a redox atmosphere. These results are similar to the findings of Sardi, et al.^21^ (2012), who found a high activity of proteinase in patients with type 2 diabetes mellitus.

Phospholipase activity on *C. albicans* strains not only determine commensal colonization, but also provide potential pathogenic yeasts^11^. Studies have reported that 30 - 100% of the oral *C. albicans* isolates produce phospholipases, with varying degrees of enzyme activity^25^. In this study, this enzyme was detected in 53.2% of *Candida albicans* strains when incubated in a reduced oxygen atmosphere. The anaerobic condition showed 33.5% activity.

Our analysis showed no statistically significant difference for the hemolysin activity in diabetic and normoglycemic patients in both tested atmospheres. Manns, Mosser and Buckley^13^ (1994) determined the conditions under which *C. albicans* can show hemolytic activity and found that hemolysis is not observed when glucose is not available in the culture medium.

The cell surface hydrophobicity was evaluated under anaerobic and redox conditions, with the results indicating greater hydrophobicity in anaerobic conditions. The results of this study indicated that 72% of the isolates were highly hydrophobic under anaerobic atmospheres and 64.3% under the redox atmospheres. Hydrophobic interactions may be important in the tissue invasion of the mycelia phase of yeast cells^21^.

Our findings follow the same trend of the authors Siqueira and Sen^23^ (2004), and Gomes, et al.^8^(2010), who investigated the importance of filamentous fungi in root canals of teeth with pulp necrosis and periradicular lesions, which may participate in the etiology of periradicular diseases.

The duration of diabetes and quality of metabolic control are related to the development of diabetic complications. Poorly controlled diabetes has been associated with high incidence of *Candida* infections and such situation may suggest difficulty in treatment^4^. *C. albicans* seems to be present more frequently in the root canals of obturated teeth in which treatment has failed^22^.

## Conclusion

The prevalence of *Candida albicans* was higher in patients with diabetes mellitus in samples collected from periodontal pockets and root canals. Strains of *Candida albicans* showed increased virulence in diabetic patients compared to normoglycemic patients. The production of proteinase and phospholipase activity were higher in diabetic patients when analyzed in redox. Hydrophobicity of the strains were higher in diabetic patients under anaerobic conditions. However, hemolytic activity was positive for both groups in both atmospheres.
